# Nesfatin-1 and Vaspin as Potential Novel Biomarkers for the Prediction and Early Diagnosis of Gestational Diabetes Mellitus

**DOI:** 10.3390/ijms20010159

**Published:** 2019-01-04

**Authors:** Radzisław Mierzyński, Elżbieta Poniedziałek-Czajkowska, Dominik Dłuski, Jolanta Patro-Małysza, Żaneta Kimber-Trojnar, Maciej Majsterek, Bożena Leszczyńska-Gorzelak

**Affiliations:** Chair and Department of Obstetrics and Perinatology, Medical University of Lublin, Poland, ul. Jaczewskiego 8, 20-954 Lublin, Poland; ellapc@mp.pl (E.P.-C.); p.l.casiraghi@wp.pl (D.D.); jolapatro@wp.pl (J.P.-M.); zkimber@poczta.onet.pl (Ż.K.-T.); maciek_majsterek@wp.pl (M.M.); b.leszczynska@umlub.pl (B.L.-G.)

**Keywords:** gestational diabetes mellitus, pregnancy, nesfatin-1, vaspin

## Abstract

Gestational diabetes mellitus (GDM) is considered to be one of the most frequent medical complication observed among pregnant women. The role of adipokines in the pathogenesis of GDM remains strictly unknown. Different adipokines have been studied throughout gestation, and they have been proposed as biomarkers of GDM and other pregnancy-related complications; however, there is no biomarker reported for GDM screening at present. The aim of this study was to evaluate serum nesfatin-1 and vaspin levels in GDM and non-GDM women, to characterize the correlation between these adipokines, and to assess the potential role of circulating adipokines in the prediction of risk of gestational diabetes mellitus. Serum concentrations of nesfatin-1 and vaspin were measured in 153 women with GDM, and in 84 patients with uncomplicated pregnancy by enzyme-linked immunosorbent assay (ELISA) kits, according to the manufacturer’s instructions. Circulating levels of nesfatin-1 and vaspin were significantly lower in the GDM group than in the control group. Nesfatin-1 levels were negatively correlated with vaspin levels. The results of this study point out the possible role of nesfatin-1 and vaspin as potential novel biomarkers for the prediction and early diagnosis of GDM. Further studies are necessary to evaluate the influence of nesfatin-1 and vaspin on glucose metabolism in the early stages of GDM.

## 1. Introduction

Gestational diabetes mellitus (GDM) has been defined as any degree of glucose intolerance, with beginning at or first detected at any time during pregnancy. It is one of the most frequent medical complications observed among pregnant women [1]. The incidence of GDM varies widely, from 5 to 20% of all pregnancies, depending upon the type of test chosen, the population investigated, and the methodology employed. GDM is associated with a higher risk of maternal morbidity and mortality, and adverse neonatal outcomes. The explanation of the GDM pathogenesis is important, because it may be helpful for preventing its complications. Women with GDM are also at significantly higher risk for developing type 2 diabetes mellitus (T2DM) after pregnancy, and at long-term risk of obesity and glucose intolerance in their offspring [2].

Pregnancy is characterized by a progressive decrease in insulin sensitivity [3]. Decreased insulin sensitivity and increased insulin resistance in women before fertilization, inadequate insulin production after conception, and β-cell functional impairment are all believed to be the risk factors for GDM development [4].

It may be supposed that the majority of GDM patients could have a chronic β-cell dysfunction. Buchanan et al. have suggested that this is in accordance with the fact that GDM is more frequently diagnosed in older pregnant patients (over 30 years old), who have had multiple pregnancies, and who are obese [5].

A large number of metabolic modifications observed during pregnancy are influenced by a variety of bioactive peptides, collectively known as adipokines. Adipokines are mostly produced by adipocytes [4]. It has been reported that they are also produced by the placenta, and that the serum levels of these chemical messengers might alter during pregnancy [3].

It has been found that adipokines play a significant role in maternal–fetal demands, and actively participate in various metabolic processes. The dysregulated production or secretion of adipokines in the late second and third trimester of pregnancy is implicated in insulin resistance [6]. The importance of adipokines in the pathogenesis of GDM remains unclear. Some of them have been analyzed as potential markers for GDM, but currently, none of them currently have been confirmed as a biomarker for GDM screening [6].

Nesfatin-1 is an 82 amino acid (aa) polypeptide derived from the protein precursor nucleobindin-2 (NUCB2), which is encoded by the *NUCB2* gene. Specific convertase enzymes, such as PC3/1 and PC2, change NUCB2 to active isoforms: nesfatin-1, nesfatin-2, and nesfatin-3; between 1–82 aa, 85–163 aa, and 166–396 aa, respectively. The exact information on the biological activity of nesfatin-2 and nesfatin-3 is not available [7]. The nesfatin-1 molecule consists of a few discrete domains: a signal peptide at the N-terminal, a Leu/Ile-rich domain, a DNA-binding domain, the signal for nuclear directing, and two motifs for the Ca2+-EF-hand and leucine zipper domain [7]. The structure of the nesfatin-1 molecule is also tripartite; the first section, which begins from the N-terminal and is 23 amino acids long, is called N23; the central fragment from 23 to 53 is named M30, and the section from 53 to 82 near the carboxyl terminus is called C29 [8].

Nesfatin-1 has been identified as the adipokine that is involved in metabolic processes, including glucose homeostasis, and it has a glucose-dependent insulinotropic effect. Nesfatin-1 is expressed in a few regions of the hypothalamus, and it has been proven to affect the feelings of hunger and satiety, and body weight, and play a key role in regulating food intake [9]. The concentration of nesfatin-1 increases after feeding, and the expression of nesfatin-1 was found in reward-related areas, and this suggests that nesfatin-1 can also play a role in hedonic feeding [10]. It should be emphasized that nesfatin-1 has also been featured as a regulator of blood glucose concentrations. However, the exact intracellular mechanism of nesfatin-1’s effect on decreasing blood glucose concentration is not well recognized. Moreover, nesfatin-1 has been suggested to be involved in type 2 diabetes mellitus (T2DM) pathogenesis by stimulating free acid utilization. A significant decrease in fasting plasma nesfatin-1 levels in T2DM and polycystic ovary syndrome (PCOS) patients has also been confirmed. This could be caused by impaired insulin sensitivity and suggests that nesfatin-1 might be inhibited by insulin resistance, hyperglycemia, and hyperinsulinemia [11].

Since the data concerning the levels and the role of nesfatin-1 in gestational diabetes mellitus (GDM) are ambiguous, one of the aims of this study was to evaluate the levels of nesfatin-1 in a group of pregnant women with GDM.

Vaspin (visceral adipose tissue-derived serpin; serpinA12) was identified as member of the serine protease inhibitor family [12]. Vaspin was isolated for the first time from Otsuka Long-Evans Tokushima Fatty (OLETF) rats—a rodent model for insulin resistance and abdominal obesity [13]. It is mainly expressed in visceral adipose tissue, but it might also be detected in the stomach, liver, pancreas, hypothalamus, serum, saliva, gingival fluid, and cerebrospinal fluid [14]. Caminos et al. have proven that vaspin is also expressed in human and rat placentas. In the first trimester of pregnancy, it was found in humans in syncytio- and cytotrophoblasts, but only in syncytiotrophoblasts in the third trimester [15]. Vaspin expression depends on the gestational age, and its concentration increases progressively throughout pregnancy, reaching the maximum at the end of gestation [15].

The data on vaspin serum concentrations tend to be ambiguous. Patients with extremely high vaspin levels were reported by Hida et al. and Teshigawara et al. in Caucasian and Asian ethnic groups, respectively [16,17]. Teshigawara et al. have described the minor allele sequence (A) of *rs77060950*, which could be responsible for this phenomenon [17]. Breitfeld at al. have identified several single nucleotide polymorphisms (SNPs) on the 14th chromosome in the vaspin locus that are connected with vaspin levels, which are probably the most likely reason for variations of serum vaspin concentrations [18]. One of these polymorphisms—SNP rs2236242, has a significant correlation with genotype AA of T2DM [19]. Vaspin influences glucose and lipid metabolism; additionally it works as an insulin sensitizer with anti-inflammatory and anti-atherogenic effects [20,21]. Kloting et al. have observed increased vaspin messenger RNA (mRNA) expression in the adipose tissue of obese and T2DM patients, which suggests the possibility of a compensatory mechanism for severe insulin resistance [22]. It has been published that central vaspin administration led to decreased food intake and it had sustained blood glucose-lowering effects [23]. The role of vaspin in the pathogenesis of GDM remains poorly understood, and the correlation between circulating vaspin levels and the development of GDM remains quite controversial.

The aim of this study was to determine serum nesfatin-1 and vaspin levels in GDM and healthy pregnant women, to assess the correlation between these adipokines, and to evaluate the possible role of circulating adipokines in the prediction of the risk of gestational diabetes mellitus.

## 2. Results

The women from both groups (GDM and control) were not significantly different with respect to age, gravidity, estimated fetal weight, gestational age, and body mass index (BMI) at sampling. Pre-pregnancy BMI was significantly higher in the GDM group than in the control one (23.71 ± 2.64 vs. 22.81 ± 2.05 kg/m^2^, *p* < 0.05) (Table 1).

The glucose levels during oral glucose tolerance test (OGTT) fasting at the first and second hours of the test, were significantly higher in the GDM group than in the control group (0′: 5.20 (93.66) ± 0.38 (6.99) vs. 4.46 (80.32) ± 0.39 (7.16) mmol/L (mg/dL), *p* < 0.00001; 1′: 10.06 (181.10) ± 1.12 (20.23) vs. 7.59 (136.70) ± 1.41 (25.43) mmol/L (mg/dL), *p* < 0.00001; 2′: 8.71 (156.88) ± 1.05 (18.94) vs. 6.72 (121.05) ± 1.19 (21.43) mmol/L (mg/dL), *p* < 0.00001) (Table 2).

**Figure 1:**
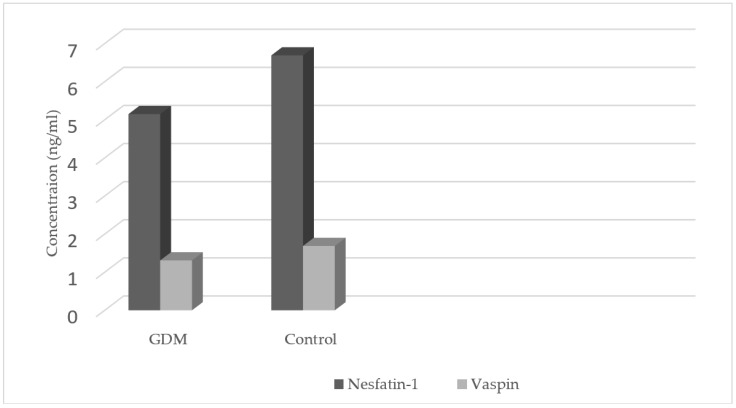
Nesfatin-1 and vaspin concentrations in the GDM and control groups.

The levels of nesfatin-1 were significantly lower in the GDM group as compared with those in the control group (5.15 ± 3.51 vs. 6.69 ± 4.21 ng/mL, *p* < 0.01) as well as vaspin concentrations (1.31 ± 0.78 vs. 1.69 ± 0.68 ng/mL, *p* < 0.0001) (Table 2, Figure 1). Nesfatin-1 levels were negatively correlated with the vaspin levels (R = −0.471, *p* < 0.0001) in the GDM group, and in the control group (R = −0.372, *p* < 0.001) (Table 3).

Pairwise correlations between nesfatin-1, vaspin levels, and clinical and demographic characteristics (maternal age; gravidity; pre-pregnancy BMI and BMI at sampling; gestational age and estimated fetal weight at sampling; glucose levels: fasting, at the first and second hours of OGTT) were determined for both groups. The correlations are presented in Table 3.

Nesfatin-1 level was positively correlated with pre-pregnancy, and at sampling BMI in the GDM group (R = 0.436, 0.369, respectively), and in the control group (R = 0.403, 0.317, respectively). A statistically significant positive correlation of nesfatin-1 level and the glucose levels: fasting, at the first and at the second hours of OGTT, were observed in patients with GDM (R = 0.382, 0.514, 0.463, respectively), and in the control group (R = 0.631, 0.565, 0.490, respectively). The nesfatin-1 level was also positively correlated with the gestational age at sampling (R = 0.274) in the control group.

The vaspin level was negatively correlated with pre-pregnancy, and at sampling BMI in the GDM group (R = −0.713, −0.648, respectively) and in the control group (R = −0.613, −0.558, respectively) (Table 3). A statistically significant negative correlation of the vaspin level and the glucose levels: fasting, at the first and at the second hours of OGTT, were observed in patients with GDM (R = −0.371, −0.472, −0.575, respectively) and in the control group (R = −0.514, −0.364, −0.348, respectively). There was also a negative correlation noted between vaspin level and the age of the patients in the GDM group (R = −0.230).

The univariate linear regression model revealed that an increase of serum levels of nesfatin-1 and vaspin by 1 ng/mL decreased the incidence of the GDM by 9.85% and 47.66%, respectively (CI 95%, OR:0.9015 vs. 0.5234). In the multiple linear regression analysis in GDM patients, it was found that the adjusted R square was higher for vaspin when compared to nesfatin-1 (0.4 vs. 0.13, respectively) when independent variables such as BMI before and during pregnancy, patients’ age, gestational age, and the fetal weight were considered.

## 3. Discussion

The exact pathogenesis and pathophysiology of gestational diabetes mellitus are unknown. Various adipokines have been shown to be dysregulated in GDM. However, the role of adipokines in the pathophysiology of GDM is still unclear.

There are only a few studies that have analyzed the serum level of nestafin-1 in pregnant woman with GDM, and the data are not clear [24,25,26,27]. In our study, we observed that in patients with GDM, nesfatin-1 serum concentrations remained significantly lower than in the group of healthy pregnant women (5.15 ± 3.51 vs. 6.69 ± 4.21 ng/mL). The same observations have been presented by Aslan et al. [24]. In this study, they examined 30 women with GDM and 30 with normal glucose tolerance. The mean nesfatin-1 level was 5.5 (8.1) ng/mL in the study group, and 8.1 (23.9) ng/mL in the control group. They also noticed that cord blood nesfatin-1 concentrations were similar in the GDM and control groups, and maternal serum nesfatin-1 concentrations were positively correlated with their respective cord blood levels [24]. As we did not analyze the levels of nesfatin-1 in cord blood, we cannot fully compare our results with the afore-mentioned findings. A study conducted by Ademoglu et al. in 40 patients with GDM, at a gestational age of between 24–28 weeks, which was similar to the gestational age of the patients enrolled in our study, proved that maternal serum concentrations of nesfatin-1 were lower compared to healthy controls (7.9 (2.8) vs. 11.2 (7.7) ng/mL) [25]. In the presented study, we analyzed nesfatin-1 levels in 153 GDM patients, and we used ELISA kits from different companies, but the results were very similar to these two studies.

Kucukler et al. have noticed that nesfatin-1 concentration was lower, and insulin level was higher in the GDM group than in the control one. They have observed a negative correlation between nesfatin-1 levels and weight, BMI, the fasting glucose level and at the first hour of the 50 g OGTT, with a homeostasis model assessment of insulin resistance (HOMA-IR). Interestingly enough, they have also found a positive correlation between nesfatin-1 and insulin levels. These findings could be suggestive of a potential role of nesfatin-1 in the pathophysiology of GDM. Therefore, they are of the opinion that the evaluation of nesfatin-1 may be useful as an early biomarker of GDM development [26].

The opposite results have been published by the group of Zhang et al. The authors demonstrated that the maternal serum and cord blood concentrations of nesfatin-1 in GDM patients were higher compared to healthy patients. The authors have concluded that nesfatin-1 is closely related to obesity and insulin resistance in pregnancy [27].

GDM and T2DM, and impaired glucose tolerance (IGT) share many common pathophysiological mechanisms such as insulin resistance and hyperinsulinemia, and all of these conditions are more often observed in overweight and obese individuals. The results of studies assessing the levels of nesfatin-1 in patients with T2DM and ITG are similarly equivocal as in patients with GDM. The observations of Algul et al. and Li et al. proved that fasting nesfatin-1 levels were decreased in T2DM and metabolic syndrome patients, compared to healthy adults and patients with T1DM [11,28]. In contrast, there was some evidence that patients with T2DM and IGT had increased levels of nesfatin-1 [29]. Zhai et al., in a meta-analysis of seven studies including 328 type 2 diabetes patients and 294 control subjects, observed no obvious differences in circulating nesfatin-1 levels. However, subgroup analysis revealed higher nesfatin-1 concentrations in newly diagnosed type 2 diabetes patients (2.01 (0.64) vs. 1.35 (0.43) μg/L and 1.91 (0.79) vs. 1.41 (0.58) μg/L), and significantly lower nesfatin-1 concentrations in type 2 diabetes patients receiving antidiabetic treatment (1.26 (0.319) vs. 1.51 (0.467) μg/L, and 0.99 (0.548) vs. 1.48 (0.816) μg/L). This analysis supports a correlation between circulating nesfatin-1 levels and type 2 diabetes [30].

The discrepancy between the results presented in this paper and previously reported in the literature might be explained by the different study protocols and patient selection processes, including: gestational age when samples were obtained—the second or third trimester, the type of GDM—treated only with diet or with insulin, which could be suggestive of the severity of metabolic disturbances, the gestational age at the diagnosis of GDM—the first (possible IGT from the pre-pregnancy period) or second trimester (“typical” gestational diabetes), the BMI value—from the pre-pregnancy period or at enrollment to the studies.

In this study, the significant correlations between the nesfatin-1 levels and the BMI values before pregnancy, and BMI values during pregnancy at blood sample collection in both groups were observed. The results of our study are comparable with the report of Ramanjaneya et al., who revealed that human plasma nesfatin-1 levels correlated positively with increasing BMI [31]. We have found a positive significant correlation between nesfatin-1 levels and glucose concentrations at any point of the oral glucose tolerance test in both groups, which confirms previous observations that plasma nesfatin-1 is positively correlated with fasting glucose concentrations [31].

Additionally, in the control group, a positive correlation between nesfatin-1 levels and gestational age the sampling has been demonstrated. This observation may suggest that the increase in nesfatin-1 concentrations as pregnancy develops could be one of the postulated mechanisms for preventing gestational diabetes mellitus development, regardless of maternal BMI. In the presented study, the patients with GDM and healthy patients had similar BMI values when blood samples were taken, but the patients with GDM had significantly lower nesfatin-1 blood concentrations. This observation might indicate the presence of impaired nesfatin-1 synthesis or release in patients with GDM, and its involvement in GDM development.

The impact of nesfatin-1 on the regulation of glucose metabolism still remains to be resolved. Previous research conducted on animal models has demonstrated the hypoglycemic properties of nesfatin-1: the intravenous administration of nesfatin-1 resulted in a significant decrease in glucose concentrations in ob/ob mice by increasing insulin action [32]. Su et al. have suggested that the decreased nesfatin-1 concentration during pregnancy and the reduced insulinotropic effect can cause the dysregulation of insulin release in GDM patients [32]. It has been shown that the nesfatin-1 release by islet β-cells exposed to glucose was increased [33]. Additionally, it has been observed that nesfatin-1 enhanced the expression of preproinsulin and glucose-induced insulin secretion via Ca2+ influx through L-type channels, irrespective of protein kinase A (PKA) and phospholipase A2 (PLA2). Hence, impaired nesfatin-1 synthesis or release might be involved in the development of metabolic conditions, mainly T2DM [34,35].

It has also been found that insulin intensifies the expression of nesfatin-1 in adipose tissue [31]. So, it could be concluded that the impact of nesfatin-1 on glucose metabolism could be entirely insulin-dependent [36]. The results of the study of Yang et al. have revealed a significant decrease in hepatic glucose synthesis, and enhanced muscle glucose uptake after nesfatin-1 infusion to the third cerebral ventricle. Additionally, nesfatin-1 increased the insulin receptor (InsR)/insulin receptor substrate-1 (IRS-1)/AMP-dependent protein kinase (AMPK)/Akt kinase (Akt)/target of rapamycin complex (TORC) 2 phosphorylation, which brought about the intensification of Fos immunoreactivity in the hypothalamic nuclei, which are involved in glucose metabolism. The authors of this report are of the opinion that nesfatin-1, through a neural-mediated mechanism, could enhance insulin sensitivity by decreasing gluconeogenesis and intensifying peripheral glucose uptake [37]. Recapitulating the reported data, nesfatin-1 may influence glucose homeostasis via mechanisms that intensify insulin release and that increase its sensitivity.

According to the results of our study, it seems that decreased levels of nesfatin-1, which is believed to be an insulinotropic adipokine in patients with GDM, might be one of the mechanisms that are responsible for the development of gestational diabetes mellitus.

Additionally, we evaluated serum vaspin levels in GDM and healthy pregnant women. It has been suggested that this adipokine may be involved in the regulation of endogenous glucose metabolism. The administration of recombinant vaspin to obese rodents enhances glucose tolerance and insulin sensitivity [38]. The precise mechanisms and correlations between vaspin secretion, and the decrease of glucose metabolism and insulin sensitivity remains unknown. Vaspin can inhibit a protease that is involved in the decomposition of a hormone or molecule, and cause, directly or indirectly, a decrease in glucose levels [39]. Higher vaspin levels reduce the cytokine-induced activation of intracellular and pro-inflammatory transcription factor nuclear factor kappa B (NF-κB) signaling cascades in 3T3-L1 cells by the inhibition of TNF-α and IL-1, the reduction of Toll-like receptor 4 (TLR4) expression, and the activation of the AMP-activated protein kinase (AMPK) pathway [40,41,42,43]. This might be a mechanism of compensation, in which white adipose tissue is a target that is activated in response to decreased insulin sensitivity [13,40]. Vaspin also inhibits the TNFα-induced expression of intracellular adhesion molecule-1 (ICAM-1), vascular adhesion molecule-1 (VCAM-1), E-selectin, and monocyte chemoattractant protein-1 (MCP-1), by blocking NF-κB and preventing the production of reactive oxygen species [42].

Circulating vaspin levels are likely to reflect its expression in adipose tissues [44]. However, it has been noticed that vaspin concentrations were significantly reduced after placental delivery, suggesting that the placenta probably also influences the circulating levels of this adipokine [45].

The data concerned with vaspin serum concentrations during pregnancy appear to be ambiguous, and the association between this adipokine level and the development of GDM is controversial. We have found lower vaspin levels in the GDM group than in the control group (1.31 ± 0.78 vs. 1.69 ± 0.68 ng/mL). In the research study published by Giomisi et al., 106 pregnant women between 24 and 30 weeks of gestation were compared with 106 non-pregnant controls. They measured vaspin, adiponectin, glucose, insulin concentrations, and lipid parameters, and used the quantitative insulin sensitivity check index (QUICKI). They noticed significantly decreased levels of vaspin (26 (15) vs. 32 (18.5) ng/mL, respectively), and increased insulin levels in pregnant patients, in comparison with non-pregnant women. There was a positive correlation between vaspin and adiponectin, and a negative correlation with lipid parameters (triglycerides, total cholesterol, low-density lipoproteins) [46]. In comparison with our study, the range of values is different, but the trend is similar. In our study we used a different ELISA kit with higher sensitivity (0.021 ng/mL vs. 2.26 ng/mL in Giomisi group study).

Huo et al. analyzed vaspin plasma levels and its placental expression in 30 pregnant GDM patients, and compared this to 27 women with uncomplicated pregnancies. Venous blood samples were collected before caesarean section, and three days after delivery, after 8 h of fasting. Serum vaspin levels during pregnancy were significantly decreased in the GDM group compared to the control group (0.49 ± 0.24 vs. 0.83 ± 0.27 ng/mL, respectively). Three days after cesarean section, vaspin concentrations were significantly lower than the levels before delivery, in the GDM group. However, there were no statistically significant differences between placental mRNA levels of vaspin and protein expression in both groups [47].

The opposite results have been published by the group of Jia at al. [48] who analyzed vaspin, leptin, and adiponectin levels in pregnant patients with or without GDM, and in non-pregnant women. Vaspin serum concentrations were markedly higher in the GDM group than in other groups [48].

Also, the opposite findings were reported by Gkiomisi et al. [45]. They measured vaspin levels in women with and without GDM during pregnancy, at the second and third trimesters, and after delivery. Serum vaspin concentrations were decreased significantly postpartum, in comparison with the second and third trimesters in all groups, and were lower in the control group than in the GDM group [45].

Mm et al. have compared serum vaspin concentration, and mRNA and protein levels of vaspin in adipose tissue, in patients with GDM and in healthy controls. They examined 73 pregnant patients: 37 with gestational diabetes mellitus and 36 with the normal glucose tolerance. Vaspin serum levels, and mRNA and protein levels were significantly increased in the GDM group [49].

On the other hand, Stepan et al. have suggested that serum vaspin concentration was not significantly different between GDM and non-GDM patients, and that no association between serum vaspin concentration and insulin resistance was observed [50].

As for nesfatin-1 results, the discrepancy between our results and other publications might be explained by the reasons mentioned above.

In non-pregnant patients, Yang et al. have found that the serum vaspin level was significantly higher in the T2DM group than in control one, and in the T2DM with macrovascular complications group. They have also noticed that vaspin concentrations were significantly higher in females compared to males [51].

Kloting et al., comparing BMI-, age-, and gender-matched insulin-sensitive versus insulin-resistant healthy obese patients, found very small differences in vaspin levels between these groups, suggesting that the correlations between circulating vaspin, fat distribution, and insulin sensitivity are more complex than assumed, and can be influenced by the other unknown factors [52].

In our study, a strong negative correlation between nesfatin-1 and vaspin levels was observed in both groups. To our best knowledge, this is the first study comparing jointly circulating levels of nesfatin-1 and vaspin in the same group of patients with GDM, and healthy pregnant women. So, in the literature, there are no data about the possible mechanisms of such a correlation, and the clinical implications. We could hypothesize that this finding can also confirm the potential role of these adipokines in the pathogenesis of GDM. However, the physiological and pathological significance of these findings requires further elucidation.

The univariate linear regression model revealed that an increase of nesfatin-1 and vaspin serum levels by 1 ng/mL decreased the incidence of GDM by 9.85% and 47.66%, respectively (CI 95%, OR: 0.9015 vs. 0.5234). Interestingly, the results suggest that serum levels of nesfatin-1 and vaspin are promising for GDM prediction; however, we are aware that the evaluation of these substances in the second trimester of the pregnancy (simultaneously with OGTT) is limiting in the context of finding potential markers for GDM.

In the multiple linear regression analysis we observed that the adjusted R square was higher for vaspin when compared to nesfatin-1 (0.4 vs. 0.13, respectively) when independent variables such as BMI before and during pregnancy, patients’ age, gestational age, and fetal weight were considered. The general interpretation of these results suggests that the serum level of vaspin may independently predict GDM in approximately 40% of women in contrast to nesfatin-1 levels, which may predict only 13% of GDM in the analyzed group of patients. However, we have to remember that the aim of our study was not to define the cut-off levels of nesfatin-1 and vaspin in women at 24–28 weeks of gestation. In all pregnant patients between 24 and 28 weeks of pregnancy, a 75 g glucose tolerance test is performed, with a very high detection rate. The aim of our study was to evaluate the potential and possible roles of circulating adipokines in the prediction of risk of gestational diabetes mellitus, and the further prospective analysis of these markers in the first trimester of pregnancy, before the time at which the OGTT is performed.

An inverse correlation between vaspin levels and glucose concentrations at any point of the oral glucose tolerance test was observed in the GDM group, and in the control one. We also noticed significant inverse correlations between vaspin levels and pre-pregnancy, and at-sampling BMI, in both groups. In the presented study, the patients with GDM and the healthy patients had similar BMI values when the blood samples were taken, but they had significantly lower vaspin blood concentrations. This observation might indicate the presence of impaired vaspin synthesis or release in patients with GDM, and its involvement in GDM development. 

On the other hand, Gkiomisi et al. have suggested that vaspin would rather be a marker of maternal adipose tissue, than an active part of glucose and lipid metabolism [45]. We observed an inverse correlation between vaspin and BMI, so we could hypothesize that vaspin is not a marker of adipose tissue, and that other mechanisms are involved in the regulation of vaspin levels.

It has been published that modifications in the vaspin gene are responsible for its compensatory effects on the metabolic abnormalities that might be observed in obesity. Nakatsuka et al. have revealed that vaspin-transgenic mice were protected from diet-induced obesity, abnormal glucose tolerance, and fatty liver, while mice with vaspin deficiency developed glucose intolerance, due to the upregulation of endoplasmic reticulum (ER) stress markers [53].

Mm et al. analyzed the correlations between vaspin levels and fasting insulin levels, and a homeostasis model assessment of insulin resistance (HOMA-IR), and birth-weight, in the patients with GDM and control patients. Serum vaspin concentration was not significantly associated with fasting insulin concentrations and HOMA-IR, but a positive correlation with birth weight was noticed [49]. It has been described that serum vaspin concentrations in obese patients were positively correlated with BMI, triglycerides levels, and IR [17,44,54]. Youn et al. have noticed that in non-pregnant obese or diabetic patients, elevated blood glucose levels were positively associated with higher serum vaspin levels [44].

Several studies did not find a correlation between vaspin levels and insulin sensitivity or parameters of obesity [55,56]. Stepan et al. have noticed no correlation between vaspin levels and parameters of insulin sensitivity or lipid metabolism in pregnant patients [50]. On the other hand, Gkiomisi et al. have suggested that vaspin levels were associated with IR and TG, and negatively correlated with QUICKI at the third trimester of gestation, but these correlations were not noticed at the second trimester and in the puerperium [45].

A connection between vaspin concentrations and weight loss, and lifestyle modification has been also found, but the results were inconclusive [39,57,58,59].

The data concerned with vaspin levels and its role in the pathogenesis of metabolic disorders are limited and controversial. Thus, more research is needed, in order to define the physiological and pathophysiological significance of vaspin. As for the results of this study, it seems that the decreased levels of vaspin in patients with GDM might be one of the mechanisms that are responsible for its development. 

## 4. Materials and Methods

The study comprised 153 women with GDM, and 84 healthy pregnant women. The research study was performed in the Department of Obstetrics and Perinatology, Medical University of Lublin, Poland. All included patients signed for informed consent to participate in the study. The study protocol received approval from the Bioethics Committee of the Medical University of Lublin (No. KE-0254/117/2018). The study was performed according to the principles expressed in the Declaration of Helsinki.

The inclusion criteria were as follows: gestational age between the 24th and 29th weeks, the first prenatal visit before the 10th week, singleton pregnancy, and GDM first diagnosed in the current pregnancy before the completed 28th week of gestation.

Exclusion criteria from the study included: multiple pregnancy, intrauterine growth retardation, underlying disorders: pre-pregnancy diabetes mellitus, any form of hypertension, chronic renal and liver diseases, chronic infectious diseases, systemic lupus erythematosus, and antiphospholipid syndrome.

The oral glucose tolerance test (OGTT) with 75 g of glucose according to WHO standards was performed in all patients participating in the study from the 24th to 28th weeks of pregnancy. Blood glucose was determined by the glucose oxidase method. The diagnostic criteria for GDM were based on World Health Organization (2014) criteria, and a diagnosis of GDM was made if one or more of the following criteria were present: fasting glucose level 5.1–6.9 mmol/L (92–125 mg/dL), at the first hour ≥10.0 mmol/l (180 mg/dL) and at the second hour 8.5–11.0 mmol/L (153–199 mg/dL) [60]. Pregnancy and birth information, maternal and family history, and maternal age were obtained by using medical records.

Anthropometric measurements were obtained from all participants. Pre-pregnancy body mass index (BMI) was calculated as the ratio of weight (kg) divided by height (m^2^). Height was measured by using standard wall-mounted tape in a standing position. Weight was measured by using a digital scale, and the maximum weight capacity was 150 kg. The weight was taken in light clothes, and the height without shoes. BMI was recalculated at the time of blood sampling.

The samples for research tests were taken at the same time as the blood samples for routinely performed laboratory tests. Serum concentrations of nesfatin-1 and vaspin were obtained at 24–29 weeks of gestation. The blood was delivered to the laboratory within 20 min, centrifuged (2000 g/min for 10 min at 4 °C) and the sera were aliquoted and stored at −70°C until assayed.

The nesfatin-1 concentration was measured by an enzyme-linked immunosorbent assay technique (Human Nesfatin-1 Elisa, BioVendor R&D Products, Brno, Czech Republic), as well as the vaspin concentration (Human Vaspin, BioVendor R&D Products, Czech Republic), according to the manufacturer’s instructions. The limit of detection of nesfatin-1 is 0.021 ng/mL. The intra- and inter-assay coefficients of variation (CVs) were 2.75% and 5.43%, respectively. The sensitivity of the vaspin ELISA assay was 0.01 ng/mL, while the intra-assay and inter-assay coefficients of variation were 6.5% and 5.8%, respectively.

The study group was compared with the control one as for: maternal age, gravidity gestational age at the entry to the study, pre-pregnancy BMI, BMI at sampling, estimated fetal weight (EFW) at sampling, and glucose levels at each hour of OGTT, as well as nesfatin-1 and vaspin concentrations. Correlations between nesfatin-1, vaspin, and BMI, and maternal age, gravidity, EFW, and glucose levels at each hour of OGTT were investigated.

The obtained data were assessed by the one-way analysis of variance (ANOVA) followed by Tukey’s post hoc test (Statistica, v. 12, StatSoft, Inc., Tulsa, OK, USA). The Shapiro–Wilk test for normal distribution of data and the one-tailed Student’s *t*-test, or (in unequal variance) the Cochran–Cox test (absence of normal distribution and nonparametric data), and the Mann–Whitney *U* test were performed. The results with normal distribution are presented as the means ± standard deviation (SD). To assess the correlation of the data, Pearson’s and Spearman’s correlation tests were performed. Logistic regression models were used for calculations of odds ratios (ORs) with 95% confidence intervals (CIs), predicting gestational diabetes mellitus based on nesfatin-1 and vaspin concentrations. To predict the diagnostic values of the nesfatin-1 and vaspin as the dependent variables, multiple linear regression analysis was performed. Herein, *p* < 0.05 was considered as a statistically significant difference.

## 5. Conclusions

Gestational diabetes mellitus is a problem that affects a significant number of pregnant women. It could be postulated that deficiencies of nesfatin-1 and vaspin could be one of the mechanisms involved in GDM development. In the presented study, patients with GDM were characterized by lower levels of nesfatin-1 and vaspin compared to healthy pregnant women, which might be the result of impaired synthesis or release of these adipokines. In our study, strong negative correlations between nesfatin-1 and vaspin levels was observed in both groups. However, the physiological and pathological significance of these findings requires further elucidation.

The results of this study point out the possible role of nesfatin-1 and vaspin as potential novel biomarkers for the prediction and early diagnosis of GDM.

The limitations of the study have to also be acknowledged. The number of patients was relatively small. Thus, the analysis of the results may be underpowered. Further studies are necessary to evaluate how nesfatin-1 and vaspin affect glucose metabolism in the early stages of GDM, and whether these adipokines can be adopted as its biomarkers in the first trimester of pregnancy, before the time when the OGTT is performed.

## Figures and Tables

**Table 1:** Characteristics of the study population (mean and standard deviation).

Variable	GDM Group *n* = 153	Control Group *n* = 84	*p*
Maternal age (years)	27.59 (4.87)	27.23 (4.67)	NS
Gravidity	1.86 (0.88)	1.93 (0.95)	NS
Pre-pregnancy BMI (kg/m^2^)	23.71 (2.64)	22.81 (2.05)	*p* < 0.05
BMI at sampling (kg/m^2^)	26.63 (2.11)	26.13 (1.71)	NS
Estimated fetal weight at sampling (g)	920.0 (187.8)	961.2 (168.5)	NS
Gestational age at sampling (weeks)	26.54 (1.41)	26.81 (1.26)	NS

GDM: gestational diabetes mellitus; BMI: body mass index; *p*: statistical significance; NS: statistically not significant.

**Table 2:** Nesfatin-1, vaspin and glucose levels in GDM and control group (mean and standard deviation).

Variable	GDM Group *n* = 153	Control Group *n* = 84	*p*
Nesfatin-1 (ng/mL)	5.15 (3.51)	6.69 (4.21)	*p* < 0.01
Vaspin (ng/mL)	1.31 (0.78)	1.69 (0.68)	*p* < 0.0001
Glucose (mmol/L)			
0′	5.20 (0.38)	4.46 (0.39)	*p* < 0.00001
60′	10.06 (1.12)	7.59 (1.41)	*p* < 0.00001
120′	8.71 (1.05)	6.72 (1.19)	*p* < 0.00001

GDM: gestational diabetes mellitus; *p*: statistical significance; NS: statistically not significant.

**Table 3:** Correlation between nesfatin-1, vaspin levels, and maternal age, gravidity, body mass index (BMI), estimated fetal weight (EFW), gestational age and glucose levels in OGTT in patients with the GDM and control groups.

Variable	GDM				Control			
	Nesfatin-1		Vaspin		Nesfatin-1		Vaspin	
	R	*p*	R	*p*	R	*p*	R	*p*
Nesfatin-1			−0.471	*p* < 0.0001			−0.372	*p* < 0.001
Maternal age (years)	−0.261	NS	−0.230	*p* < 0.01	0.060	NS	−0.138	NS
Gravidity	−0.018	NS	−0.127	NS	0.138	NS	−0.022	NS
Pre-pregnancy BMI (kg/m^2^)	0.436	*p* < 0.0001	−0.713	*p* < 0.0001	0.403	*p* < 0.001	−0.613	*p* < 0.0001
BMI at sampling (kg/m^2^)	0.369	*p* < 0.001	−0.648	*p* < 0.0001	0.417	*p* < 0.001	−0.558	*p* < 0.001
Estimated fetal weight at sampling (g)	0.001	NS	−0.004	NS	0.201	NS	0.071	NS
Gestational age at sampling (weeks)	0.007	NS	−0.040	NS	0.274	*p* < 0.05	0.049	NS
Glucose								
0′	0.382	*p* < 0.001	−0.371	*p* < 0.01	0.631	*p* < 0.0001	−0.514	*p* < 0.001
60′	0.514	*p* < 0.0001	−0.472	*p* < 0.001	0.565	*p* < 0.0001	−0.364	*p* < 0.01
120′	0.463	*p* < 0.0001	−0.575	*p* < 0.001	0.490	*p* < 0.0001	−0.348	*p* < 0.01

GDM: gestational diabetes mellitus; BMI: body mass index; R: Spearman correlation’s coefficient; *p*: statistical significance; NS: statistically not significant.
